# The potential of lactic acid bacteria in mediating the control of plant diseases and plant growth stimulation in crop production - A mini review

**DOI:** 10.3389/fpls.2022.1047945

**Published:** 2023-01-13

**Authors:** Nur Sulastri Jaffar, Roslina Jawan, Khim Phin Chong

**Affiliations:** ^1^ Faculty of Science and Natural Resources, Universiti Malaysia Sabah, Sabah, Malaysia; ^2^ Horticulture Research Centre, Malaysian Agricultural Research and Development Institute (MARDI), Selangor, Malaysia

**Keywords:** lactic acid bacteria, phytopathogens, biological control agent, plant disease management, plant growth

## Abstract

The microbial diseases cause significant damage in agriculture, resulting in major yield and quality losses. To control microbiological damage and promote plant growth, a number of chemical control agents such as pesticides, herbicides, and insecticides are available. However, the rising prevalence of chemical control agents has led to unintended consequences for agricultural quality, environmental devastation, and human health. Chemical agents are not naturally broken down by microbes and can be found in the soil and environment long after natural decomposition has occurred. As an alternative to chemical agents, biocontrol agents are employed to manage phytopathogens. Interest in lactic acid bacteria (LAB) research as another class of potentially useful bacteria against phytopathogens has increased in recent years. Due to the high level of biosafety, they possess and the processes they employ to stimulate plant growth, LAB is increasingly being recognized as a viable option. This paper will review the available information on the antagonistic and plant-promoting capabilities of LAB and its mechanisms of action as well as its limitation as BCA. This review aimed at underlining the benefits and inputs from LAB as potential alternatives to chemical usage in sustaining crop productivity.

## 1 Introduction

According to the Food and Agriculture Organization (FAO) of the United Nations (UN), farmers will need to produce 70 to 100 percent more food to meet the demand of the predicted growing population of 9.3 billion people by 2050 (UNDP, 2021). Apart from the growing population, depletion of natural resources, climate change, and the emergence of new pests and diseases are among the several factors that have a negative impact on agricultural production and productivity. Various pests and diseases contribute 20 to 40 percent yearly economic losses in agricultural products by reducing crop output, degrading its quality, and contaminating food with hazardous compounds (Guo et al., 2013). Plant diseases caused by phytopathogens can limit crop production in a vast array of plant species worldwide, resulting in significant annual losses on a global scale. The most common infectious plant diseases are caused by pathogenic organisms such as fungi, bacteria, viruses, protozoa, insects, and parasitic plants. Wilting, spotting (necrosis), mold, pustules, rot, overgrowth (hypertrophy), distortion (mummification), staining (discoloration), and destruction of afflicted tissue are the symptoms of plant diseases (Nazarov et al., 2020).

Plant diseases can be mitigated in a variety of ways, including cultural, physical, chemical, and behavioral practices. Growers typically rely on the use of chemical treatments, such as fungicides and bactericides, which have been in widespread use for more than a century but none of them are sustainable (Malik et al., 2021). On the other hand, the use of these synthetic chemical treatments in agriculture has been linked to causing numerous issues such as the development of resistance in pathogen populations, detrimental impacts on human health, loss of beneficial soil microbes, the introduction of leftover harmful material into the food chain, and decreased in biodiversity of microorganisms (Sindhu et al., 2016). Efforts to find alternatives to chemical treatments have been bolstered by the understanding that this method of treating plant diseases is suboptimal at best or prohibited by regulation. In addition, due to the growing demand for safety and quality of food production, the search to find alternatives with an eco-friendlier approach has become a priority. The widespread use of agrochemicals can be replaced with a method that is less hazardous. Thanks to the development of microbial biocontrol agents. The term “biological control” refers to the practice of reducing the prevalence of plant disease through the application of naturally occurring organisms, such as beneficial microorganisms or their by-products or extracts from plants or animals (Sundin et al., 2016). Their mechanisms of action toward the target pathogen can be varying either directly or indirectly, such as competition, predation, antibiosis, induced host resistance, or by the activity of lytic enzymes. Over the past decades, numerous research has been published on the potential application of beneficial microorganisms as biological control agents (BCAs) against plant pathogenic bacteria, and strains from the genera *Pseudomonas*, *Burkholderia*, *Streptomyces*, *Bacillus*, and *Trichoderma* are very well-known for their antimicrobial capability and synthesis of a wide range of bioactive compounds (Alexander and Phin, 2014; Haryadi et al., 2019; Lim et al., 2019; Alexander et al., 2021).

Interest in lactic acid bacteria (LAB) research as another class of potentially useful microorganisms against phytopathogens has increased in recent years. The application of LAB for plant protection and plant growth stimulation first appeared in the 1980s by Visser et al. (1986) and Higa and Kinjo (1989). It has been demonstrated that LAB has the ability to produce compounds that are effective in the management of a wide range of bacterial and fungal phytopathogens (Gajbhiye and Kapadnis, 2016; Daranas et al., 2019; Duha and Abdullah, 2021). Moreover, its long and widespread use history in food processing allows researchers to understand the physiological processes and bioactive substances being produced. This resulted in its being given the generally regarded as safe (GRAS) status with few exceptions (Goldstein et al., 2015), meaning that its use in edible crop cultivation poses no health concerns to humans. This paper will review the available information on the antagonistic and plant-promoting capabilities of LAB and its mechanisms of action as well as its limitation as BCA. This review aimed at underlining the benefits and inputs from LAB as potential alternatives to chemical usage in sustaining crop productivity.

## 2 Lactic acid bacteria

LAB is a group of Gram-positive, catalase-negative, non-sporulating, facultatively anaerobic, rod-shaped (bacilli) or spherical (cocci) bacteria that include 6 families and 38 genera in the *Lactobacillales* order (Holzapfel and Wood, 2014). It produces lactic acid (LA) as the primary end product of saccharolytic metabolism (Hayek and Ibrahim, 2013) and has been classified into homofermentative and heterofermentative strains based on their lactic acid (LA) production. Homofermentative LAB converts sugars to lactic acid, while heterofermentative LAB produces lactic acid, ethanol or acetic acid, and carbon dioxide. The most prevalent species include *Lactobacillus*, *Lactococcus*, *Enterococcus*, *Streptococcus*, *Pediococcus*, *Leuconostoc*, *Weissella*, and *Bifidobacterium* (Abubakr and Al-Adiwish, 2017). Studies on LAB identification was done long time ago because it was important to know their properties and ensure that the strains were safe to use. Phenotypic and chemical approaches were formerly used to identify LAB. Different types of LAB activity, such as carbohydrate fermentation, hetero- or homofermentative, gas production, motility, and spore production, form the basis for these techniques (Ashmaig et al., 2009). However, LAB identification based on carbohydrate fermentation profiles is imprecise and insufficient to identify closely related strains due to their similar nutritional requirements (Perricone et al., 2014). Therefore, genomic sequencing is the most reliable way to accurately identify bacteria.

Rapid advances in molecular biology in recent years have had far-reaching effects on the discipline of microbiology allowing the use of 16S rDNA gene sequencing techniques for the identification of bacteria, including LAB. These conserved genes exhibit sufficient variation to be regarded as excellent phylogenetic markers, which can be used for identifying organisms down to the genus and species level (Chong et al., 2013; Lo and Chong, 2020). The development of whole genome sequencing (WGS) technology in recent years has also substantially accelerated the development and application of LAB resources. It enables researchers to systematically and comprehensively understand the metabolic characteristics, potential beneficial functions, and application directions of the strains based on the information contained in the whole genomes of LAB (Buron-Moles et al., 2019). In addition, WGS allows the determination of the genetic evolution and classification of LAB in a more accurate manner (Huang et al., 2020). The first complete LAB genome sequence was published in 2001 for the species *Lactococcus lactis* IL1403 (Bolotin et al., 2001). Since then, 7,055 species of LAB that includes *Lactobacillus*, *Lactococcus*, *Bifidobacterium*, and *Streptococcus thermophilus* have had genomic data (comprising whole genomes and framework genomes of varying degrees) deposited in the GenBank (Yanwei et al., 2022) enabling for a comprehensive understanding of the industrial application and metabolic characteristics of LAB. Whole genome information can reflect the safety of LAB strains by assessing genes related to drug resistance, virulence, and pathogenicity and determining whether the related genes can be transmitted horizontally (Rodríguez-Serrano et al., 2018; Toropov et al., 2020; Zhou et al., 2021). Furthermore, the genome-scale metabolic model (GSMM) can be reconstructed from whole-genome data to simulate and anticipate how bacteria will behave in each environment and to systematically direct metabolic engineering efforts. The first GSMM of LAB was generated using the genome sequence of *Lactococcus lactis* IL1403, which effectively predicted and confirmed the minimum medium for strain development and steered the metabolism to enhance diacetyl production (Oliveira et al., 2005). Other successful examples of GSMM in guiding metabolic engineering of microbial improvement in LAB have also been documented (Vinay-Lara et al., 2014; Xu et al., 2015; Kristjansdottir et al., 2019).

### 2.1 Diversity, abundance, and plant colonization attributes of LAB in plant microbiome

Lactic acid bacteria (LAB) are found virtually everywhere in the natural world, including in the phyllosphere, endosphere, and rhizosphere (Liu et al., 2014). Their abundance varies greatly from one environment to another. The genomic variation in LAB strains is largely attributable to the selective pressure exerted by these settings (McAuliffe, 2018). LAB in the phyllosphere microbiota was discovered to make up a relatively small percentage of the total bacteria detected on plant tissues, ranging between 10^2^ and 10^4^ CFU/g (di Cagno et al., 2013). It has been acknowledged that LAB may live as an endophyte in a variety of crop plants (Suzzi et al., 2018; Filannino et al., 2019) and that it can survive in seeds (Taha et al., 2019). In the rhizosphere, several LAB strains were discovered to exhibit antimicrobial properties (Fhoula et al., 2013). Carbon-richness is a major determinant of LAB abundance and variety in soils (Yanagida et al., 2006; Reyes-Escogido et al., 2010; Ain et al., 2017). Soil acidity may also play a role in the recovery of several halotolerant LAB, which are known to survive and even thrives in arid settings. Among the commonly found genera of the LAB families from plant microbiomes includes *Enterococcus*, *Lactococcus*, *Lactobacillus*, *Leuconostoc*, *Streptococcus*, and *Weissella* (Yu et al., 2020).

Plants are usually thought to be difficult settings for many microbes. The phyllosphere, for instance, is subject to fast shifts in water supply, UV radiation, oxidative stress, and temperature, and can have low nutrient contents. However, many types of plant-associated bacteria such as LAB have adapted to live and thrive on plant microbiomes despite these stressors. Some properties of LAB suggest that these bacteria could also be successful plant colonists. LAB prefers glucose, fructose, and sucrose as carbon sources for fermentative development. These sugars are found in the phyllosphere and can amount up to 12.5 μg g^−1^ leaf or 38 g/100 ml of floral nectar (Canto and Herrera, 2012). LAB are not known to metabolize hemicellulose, yet genes associated in hemicellulose breakdown such as α-glucuronidase (*algA*), polysaccharide deacetylase (*pda*), endoxylanase/endoglucanase (llkf_1370), and acetylesterase (llkf_1374), were found to be upregulated in *L. lactis* KF147 grown in a lysate of *Arabidopsis thaliana* leaves (Golomb and Marco, 2015). LAB also produces metabolites that have a direct impact on plant development (e.g. 2,3-butanediol and acetoin) (Sharifi and Ryu, 2018). This was demonstrated for *L. plantarum* WCFS1, which was found to produce plant hormone-like compounds that interfered with plant root development when incubated under slow growth, substrate-limited conditions (Goffin et al., 2010). These findings imply that, even in the absence of measurable levels of plant growth, LAB may be an important modifier of plant physiology.

A great number of LAB are also able to survive in aerobic (aerotolerant) and low water activity (osmotolerant) environments. They have the ability to eliminate reactive oxygen species (ROS) through the synthesis of NADH oxidases, superoxide dismutase, cytochrome d oxidase or nonenzymatic dismutation of hydrogen peroxide (H_2_O_2_) by Mn^2+^ (Papadimitriou et al., 2016). Osmotolerance in LAB is mostly connected with enhanced transport of compatible solutes and elevated synthesis of protease, chaperones, and peptidoglycan. Exopolysaccharides (EPS) synthesis is also linked to LAB’s ability to survive in environments with limited water (Nwodo et al., 2012). In addition, the ability of LAB to generate antimicrobial substances may also aid in its colonization in the plant microbiomes. Intra-specific genetic and phenotypic variations among LAB also provide more evidence that certain LAB strains are better adapted to plant environments than others (Strafella et al., 2021). Ayad et al. (1999) reported that plant-isolated *Lactococcus lactis* was discovered to have lower amino acid needs for growth compared to *L. lactis* isolated from other sources. Comparative genomic analysis of the *L. lactis* strains KF147 (isolated from mung bean sprouts) and IL1403 (isolated from cheese) revealed that the KF147 genome contains more genes encoding for carbohydrate metabolism (xylan and arabinose degradation), EPS biosynthesis, bacterial defense (nisin biosynthesis), and stress response (Siezen et al., 2010). *L. lactis* KF147 also grew faster and had a greater final cell density than IL1403 when cultured in *Arabidopsis thaliana* leaf lysate (Golomb and Marco, 2015).

## 3 Mechanism of action of LAB in controlling disease and stimulating plant growth

**Figure 1:**
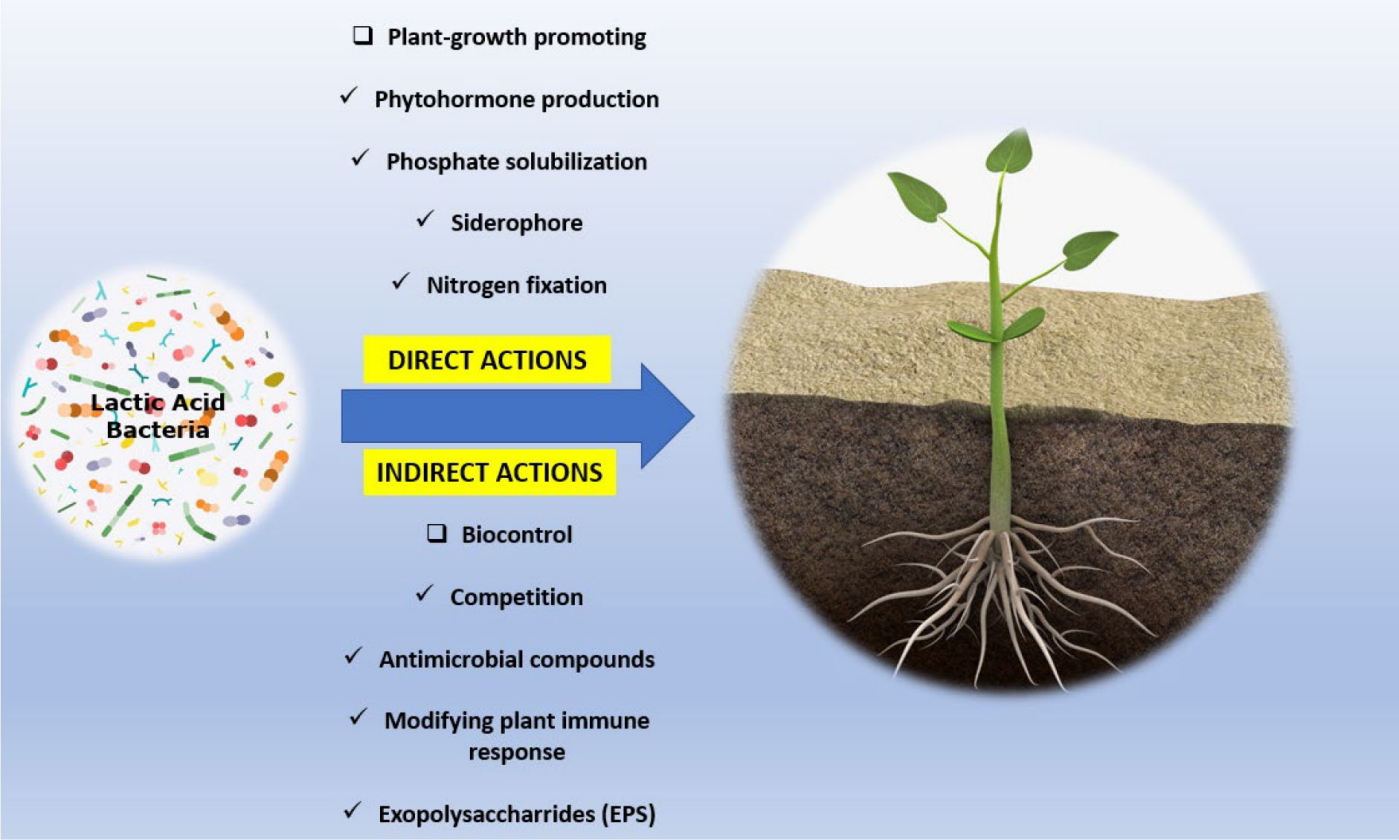
LAB mechanisms of biological control and plant-growth promoting for plant health.

The properties of some LAB species made them the potential candidate for biological control agent (BCA) (Tsuda et al., 2016; Daranas et al., 2019). Despite not having the same reputation as other groups of BCA like *Pseudomonas*, *Burkholderia*, *Streptomyces*, *Bacillus*, and *Trichoderma*, several LAB species were reported for the ability to suppress the action of phytopathogens and were also found to stimulate plant growth (Jaini et al., 2022). As illustrated in **Figure 1**, LAB can help directly with plant disease control and plant growth by modulating the uptake of important nutrients like phosphorus and potassium, fixing nitrogen, and the production of plant hormones and siderophores. Indirectly, LAB could aid in the biocontrol of phytopathogens through the production of a wide range of antimicrobial compounds including diketopiperazines, hydroxy derivatives of fatty acids, 3-phenyllactate; antibacterial bacteriocins and bacteriocin-like inhibitory substances (BLIS) organic acids, hydrogen peroxide, pyrrolidone-5-carboxylic acid, diacetyl, and reuterin (Lamont et al., 2017), modulating defense mechanism by creating systemic resistance, and decreasing pathogen iron availability. It has also been proposed that multiple mechanisms of action might be involved in the attack of LAB against phytopathogens (Sangmanee and Hongpattarakere, 2014).

### 3.1 Direct mechanisms

#### 3.1.1 Modulating the intake of nutrients and the fixation of nitrogen

Some strains of LAB can boost the availability of nutrients derived from compost and other forms of organic or inorganic matter to plants (Lamont et al., 2017). Phosphorus (P), the major macronutrient for plant growth, is mostly stored in the soil either as an organic compound or as an inorganic precipitate. Similarly, a deficit in potassium (K) which is predominantly contained in fixed form, has detrimental effects on the overall growth and yield of a plant. It was suggested by de Lacerda et al. (2016) that the presence of gene sequences encoding for two types of alkaline phosphatase—the enzymes that catalyze phosphate mineralization—allows *L. lactis* to solubilize several sources of phosphorus compound. The acidity induced by LAB, which is caused by the synthesis of organic acids, is also responsible for the solubilization of P and K, which then makes these elements available for the plant to absorb. Apart from LAB capabilities to dissolve phosphate, Giassi et al. (2016) reported that some strains of the LAB were also able to fix atmospheric nitrogen for plant consumption. Biological nitrogen fixation (BNF) is a process in which atmospheric N_2_ is transformed into ammonia and nitrate with the help of the nitrogenase enzyme complex. Higdon et al. (2020) reported that *L. lactis* isolated from mucilage microbiota of Sierra Mixe maize were recently found as diazotrophs capable of BNF. Molecular functions associated with polysaccharide catabolism, glycan-mediated host adhesion, iron/siderophore utilisation, FeMo cofactor biosynthesis (NifB), and novel oxidoreductase activities were discovered through protein domain analysis of the identified unknown genes in *L. lactis*, indicating their importance for the BNF trait.

#### 3.1.2 Phytohormones production

Plants and bacteria both generate phytohormones in very low concentrations that can influence plant growth. These phytohormones enhance root hair length and surface area, which improves plant root nutrition and water uptake (Kumar et al., 2022). The increased metabolic activity caused by phytohormones production aids in defence, normal cell function, and abiotic stress management (Khan et al., 2020). Several LAB species are capable of secreting phytohormones such as gibberellin (GA) and auxins such as indole-3-acetic acid (IAA) which play various functions in plant growth promotion (Lamont et al., 2017). According to Turaeva et al. (2021), GA_4_ and GA_7_ were detected from the culture fluid of *L. plantarum* which enhances the plant growth and development of wheat coleoptiles through the usage of HPLC-MS. However, there is still a lack of clarity behind the mechanisms of action.

### 3.2 Indirect mechanisms

#### 3.2.1 Organic acids

Organic acids have been implicated in several studies as a major mechanism by which LAB exerts its antimicrobial activity against a wide variety of target microorganisms (Tofalo et al., 2016). Lactic acid is the most common LAB metabolite, however other acids such as acetic, propionic, formic, benzoic, and PLA acids are also produced. The antibacterial impact of lactic acid is commonly believed by interfering the membrane functions of the pathogen, inhibition of active transport, reduction of intracellular pH, and inhibition of several metabolic activities, thus killing the target microorganism (Rattanachaikunsopon and Phumkhachorn, 2010). However, the generation of lactic acid and its pH lowering effect are affected by species or strain, culture mix, and growth conditions (Olaoye and Onilude, 2011). Many bacteria, fungi, and yeasts are killed off by the presence of lactic acid in its undissociated form at low pH. The degree to which various microbes are affected by lactic acid can vary widely.

#### 3.2.2 Hydrogen peroxide

Hydrogen peroxide (H_2_O_2_) is a reactive oxygen species that is also generated by LAB in the presence of oxygen. Hydrogen peroxide has a powerful oxidizing effect on microbial cells, causing irreparable damage to the fundamental molecular structures of protein involved in cellular metabolism (Sunil and Narayana, 2008). H_2_O_2_, once generated, can inhibit the development of both psychotropic and pathogenic microorganisms. Newer research, however, suggests the antimicrobial activity of H_2_O_2_ is probably limited, as bacteria only create a small amount, and that its effects are largely in conjunction with other antifungals substances (le Lay et al., 2016).

#### 3.2.3 Bacteriocin

Bacteriocins are ribosomal generated antimicrobial peptides produced by bacteria, can kill or inhibit related or unrelated bacterial strains without harming the bacteria themselves (Yang et al., 2012). Their antimicrobial modes of action are multiples including interference with cell wall development, disruption of the cytoplasmic membrane, suppression of protein synthesis, interference with the replication and transcription of DNA, and interference with the septum formation (Ahmad et al., 2017). Certain members of LAB produce bacteriocins and bacteriocin-like inhibitory substances (BLIS). The majority of LAB bacteriocins are small thermostable or large thermolabile proteins or protein complexes with antibacterial activity against other microbes, although producer cells are immune to their own bacteriocin(s) (Zacharof and Lovitt, 2012). The pH, nutrition sources, and incubation temperature have a significant impact on bacteriocin synthesis. Based on biochemical and genetic characterization, four distinct classes of LAB bacteriocins have been identified: lantibiotics (class 1); small, heat-stable nonlanthionine peptides (class 2); large heat-labile proteins (class 3) and complex bacteriocins with chemical moieties such as lipid and carbohydrate (class 4) (Hernández et al., 2005). The management of bacterial infections in economically important crops may be possible *via* bacteriocin-mediated resistance in plants, according to recent research by Rooney et al. (2020a); Rooney et al. (2020b).

#### 3.2.4 Reuterin

Several lactobacilli have been demonstrated to produce the glycerol-derived antimicrobial compound reuterin, and its production is stimulated directly or indirectly by the presence of glycerol in anaerobic conditions. LAB do not have an oxidative pathway that would allow them to use glycerol as their primary carbon source. Consequently, LAB must utilize an alternate carbon source in order to breakdown glycerol (Bergsma et al., 2022). Reuterin is a strong inhibitory chemical with broad spectrum activity that is pH independent. It is resistant to proteolytic and lipolytic enzymes and inhibits DNA replication (Singh, 2018). Reuterin has been proven to be effective against a variety of fungus, including several species of *Fusarium*, *Penicillium*, and *Aspergillus* (Vimont et al., 2019), and has been linked to the prevention of mycotoxin development in fermented foods. It also inhibits the growth of gram-positive and gram-negative bacteria, as well as enteropathogens, yeast, fungi, protozoa, and viruses. (Nes et al., 2011).

#### 3.2.5 Cyclic dipeptides

Cyclic dipeptides or cyclodipeptides (CDPs), also known as 2, 5-diketopiperazines, are the smallest cyclic peptides, and existing data indicate that bacteria produce nearly 90% of CDPs (Mishra et al., 2017). Among the cyclic dipeptides extracted from LAB with known antimicrobial properties are cyclo(Gly-Leu), cyclo(Phe-Pro), cyclo(Phe-OH-Pro), and cyclo(Phe-OH-Pro) (Leu-Leu) (Silpa and Rupachandra, 2022). Due to their stability in many environments (pH, heat, and enzymes), cyclic peptides have received a lot of interest. Cyclo(Gly-Leu) from *Lb. plantarum* VTT E-78076 was discovered to be an antifungal chemical with antifungal activity against plant fungal pathogens *Fusarium avenaceum* (Zhao et al., 2017). In spite of its antimicrobial potential, significant research is necessary to know its mode of action and range of uses.

#### 3.2.6 Fatty acids

Antimicrobial effects of hydroxy fatty acids (FAs) have been observed by (Hou and Forman, 2000; Granér et al., 2003), and they are found in a wide variety of organisms, including mammals and plants. 3-OH-FAs are found in bacteria as lipopolysaccharides or poly-hydroxyalkanoic acids. The lipopolysaccharides and polyhydroxyalkanoic acids of LAB have not been reported. Over 90% of all cellular FAs in LAB are saturated and monounsaturated FAs, and these are the ones that have been utilized for classification of distinct LAB. However, Lee et al. (1996) found that various *Leuconostoc* strains contained 2-hydroxyhexadecanoic acid and 3-hydroxyheptadecanoic acid. Unsaturated FAs can be converted into OH-FAs by LAB (Wanikawa et al., 2002), suggesting metabolic pathways for hydroxylation of FAs, albeit the precise function of 3-OH-FAs in LAB metabolism has yet to be determined. A study by (Sjögren et al., 2003) suggested that the antimicrobial effect of 4 OH-FAs extracted from *L. plantarum* MiLAB 14 is owing to the compounds’ detergent-like characteristics, which disrupt the structure of the target organisms’ cell membranes. A molecule comparable to the 3-OH-FAs discovered here, cis-9-heptadecenoic acid, partitions efficiently into the lipid bilayers of fungal membranes. This finally causes the cytoplasmic disintegration of fungal cells by increasing membrane permeability and the release of intracellular electrolytes and proteins.

## 4 Potential role of LAB in plant stress tolerance

### 4.1 Defending plant against biotic stress

The increase in biotic and abiotic stressors poses a threat to the productivity of crops. Extreme occurrences such as the advent of plant diseases and pests and the effect of climate change are becoming more common around the world. LABs have demonstrated the ability to improve crop development and productivity by developing tolerance traits to various types of stress. These bacteria have a wide range of functional characteristics and can colonize themselves in plant tissues, positively influencing plant development and survival. Numerous studies have looked into the potentiality of LAB in controlling bacterial and fungal phytopathogens from causing destruction to crops (**Table 1**). For instance, *Lactobacillus plantarum* and *Leuconostoc mesenteroides* strains were successfully screened by using *in vitro* and *in planta* assays against three bacterial pathogens affecting three different crops, namely *Pseudomonas syringae* in kiwifruit, *Xanthomonas arboricola* in Prunus, and *Xanthomonas fragariae* in strawberry (Daranas et al., 2019). They were selected due to their broad-spectrum activity in preventing all 3 pathogens from infecting their plant hosts. Also, in both semi-field and field studies, the biocontrol performance of the *L. plantarum* strains was on par with reference controls. The generation of lactic acid and a decrease in pH was partially responsible for the inhibitory mechanism observed *in vitro* and both strains had comparable rates of survival when placed on leaf surfaces. Similar broad-spectrum inhibition was observed by the species *L. paracasei* and *L. plantarum* isolated from wine fermentations (López-Seijas et al., 2019). The LAB strains were not only able to inhibit several food spoilage Gram-positive bacteria but *in vitro* studies also showed that the LAB strains had a 55-76% effect in preventing the growth of *Fusarium oxysporum* sp. *lycopersici*, a phytopathogenic fungus that causes disease in tomatoes. The efficacy of these malolactic LAB strains was very competitive when compared to the previous studies of *L. plantarum* isolated from different sources (Rouse et al., 2008; Dalié et al., 2010). It has also been shown that the plant-derived *Weissella confusa* and *Pediococcus pentosaceous* strains both have broad-range inhibitory action against fungal diseases of fruit crops. (Crowley et al., 2012a; Crowley et al., 2012b).

**Table 1:** Selected lactic acid bacteria with biological control and biostimulant properties.

Strain	Source	Pathogen/Crop	Mechanism/Effect	References
(i) Biocontrol				
*Lactobacillus plantarum*	Cucumber pickle	*Pseudomonas campestris, Ralstonia solanacearum, Xanthomonas campestris* pv. *vesicatoria, Pectobacterium carotovorum*	Organic acids	Visser et al., 1986
*Lactobacillus* sp.	Tomato rhizosphere	*Ralstonia solanacearum, Xanthomonas* *axonopodis* pv. *citri, X. campestris* pv. *vesicatoria, Erwinia pyrifoliae*, *Pectobacterium carotovorum*	None	Shrestha et al., 2009a; Shrestha et al., 2009b
*Lactobacillus plantarum*	Kimchi	*Aspergillus flavus*	3,6-bis(2-methylpropyl)-2,5-piperazinedion	Yang & Chang, 2010
*Lactobacillus* sp.	Dairy products	*Fusarium oxysporum*	SAR, antifungal metabolites	Hamed et al., 2011
*Lactobacillus plantarum*	Fermented mare milk	*Botrytis cinerea, Alternaria solani, Phytophthora drechsleri, Fusarium* *oxysporum and Glomerella cingulate*	Proteinaceous and non-proteinaceous antifungal compounds	Wang et al., 2011
*L. fermentum*	Fermented food, dairy products	*A. niger, Fusarium graminearum, A. oryzae*	Proteinaceous, PLA	Muhialdin et al., 2011; Gerez et al., 2013
*Lactobacillus plantarum*	Durian fruit	*Colletotrichum capsici*, broad spectrum	Unknown	El-Mabrok et al., 2012
*Lactobacillus plantarum*	Ginger root	*Colletotrichum capsici*, broad spectrum	Unknown	El-Mabrok et al., 2012
*Lactobacillus paracasei*	Tomato, soil	*Ralstonia solanacearum*	Unknown	Murthy et al., 2012
*W. paramesenteroides*	Fermented wax gourd	*Rhizopus stolonifera, Sclerotium oryzae, Rhizoctonia solani, Botrytis cinerea, Sclerotinia minor, Rhodotorula* sp.	Organic acids	Lan et al., 2012; Sathe et al., 2007
*Lactobacillus acidophilus*	Chicken intestine	*Fusarium* sp*., Alternaria alternata, P. paneum, Cladosporium* sp*., Rhizopus oryzae*	Organic acids	Oliveira et al., 2014; Schnürer and Magnusson, 2005
*Lactobacillus paracasei*	Tomato, soil	*Ralstonia solanacearum*	SAR	Konappa et al., 2016
*Weisella cibaria, Lactococcus lactis* subsp. *lactis*	Papaya seed	*Erwinia mallotivora*	Organic acids, hydrogen peroxide	Taha et al., 2019
*L. pentosus*	Fruit, fermented food	*A.oryzae, A. niger, Fusarium* sp.	PLA	Ouiddir et al., 2019
*Lactobacillus pentosus, Leuconostoc fallax*	Fermented vegetables	*Alternaria brassicicola*, *Xanthomonas campestris* pv. *campestris*, *Pectobacterium caratovorum*	Unknown	Lin et al., 2020
*Lactobacillus plantarum*	Yellow pithaya	*Fusarium fujikuroi*	Unknown	Valencia-Hernandez et al., 2021
*Lactobacillus acidophilus*	Mango	*C. gloeosporioides*	Antifungal compound, lytic enzyme	Ranjith et al., 2021
*Lactiplantibacillus* *plantarum*	Collection of Pure Cultures of Industrial Microorganisms ŁOCK at the Lodz University of Technology, pickled vegetables, milk	*Pectobacterium carotovorum*, *Streptomyces scabiei*, *Alternaria solani*, *Alternaria tenuissima*, *Alternaria alternata*, *Phoma exigua*, *Rhizoctonia solani*, *Colletotrichum coccodes*	Organic acids	Steglińska et al., 2022
(ii) Biostimulant				
*Lactobacillus* sp.	Rhizosphere soil of tomato	Pepper	IAA, phosphate solubilization, and biocontrol property Increased root and shoot length, root fresh weight and chlorophyll content	Steglińska et al., 2022
*Enterococcus faecium*	Rhizosphere soil of oriental melon (*Cucumis melo* L.)	Rice	Phytohormones (GA, IAA), mineral solubilization, and biocontrol property -Increased shoot and root length, plant fresh weight, chlorophyll content, nutrient uptake	Lee et al., 2015
*L. plantarum*	PGPR Corp. (Korea)	Cucumber	Succinic acid, lactic acid increased growth, nutrient availability and amino acid content	Kang et al., 2015
*Lactobacillus* sp.	Sugarcane fermentation	Citrus seedling	Nitrogen fixation, phosphate solubilization increased height, stem diameter, root and shoot weight	Giassi et al., 2016
*Enterococcus* sp.	Rhizosphere soil of grass pea	*Fusarium oxysporum* f. sp. lentis	IAA, phosphate solubilization, stress response and biocontrol property	Mussa et al., 2018
*E. faecium* LB5, *L. lactis* LB6, LB7, and LB9	Rhizosphere soil of wheat	*Fusarium graminearum*	-Phosphate solubilization and biocontrol property	Strafella et al., 2021
*Lactobacillus* sp.	Vietnamese traditional Nem chua	Peanut seed	IAA, phosphate solubilization, and biofilm formation -Increased seed germination, vigor index, plant length, and total fresh weight	Nguyen et al., 2021
*Lactobacillus* sp.	Silage and rhizosphere soil	Adzuki bean (*Vigna angularis*), Arabidopsis	3-phenyllactic acid (PLA) -Root promoting activity in Adzuki bean, promote auxin signaling pathway – increased lateral root density in Arabidopsis	Maki et al., 2021, 2022
*Weisella cibaria, Lactococcus lactis* subsp. *lactis*	Papaya seed	Papaya	Synthesis of ammonia, siderophores, and phosphate solubilization - increased the dry weight of the shoot and root of papaya plants	Jaini et al., 2022
*Lactobacillus* sp.	The aerial part of pomegranate plants	*Fusarium* sp.	Phytohormones (GA, IAA) and biocontrol property -	Abhyankar et al., 2022

Steglińska et al. (2022) reported LAB screening of ten phytopathogens related to potato including *Pectobacterium carotovorum*, *Fusarium oxysporum* and *Rhizoctonia solani*, showed a 40-90% disease reduction except for *Fusarium oxysporum* and *Fusarium sambucinum* which were not inhibited by the LAB, *Lactiplantibacillus plantarum* KB2 LAB 03. In the metabolic profiles of the LAB strains, the most abundant compounds were found to be from organic acids and ethanol. Zebboudj et al. (2020) indicated that *L. lactis* subsp. *diacetylactis* were able to inhibit *Fusarium* species of tomato crown and root rot up to 62.42% on MRS agar medium. Another *in vitro* assessment done by Valencia-Hernandez et al. (2021) showed biomass fraction of *Lactobacillus plantarum* isolated from yellow pithaya inhibit *Fusarium fujikuroi* growth by 100% over 48 hr of fermentation. In another finding by Lin et al. (2020), *Lactobacillus pentosus* and *Leuconostoc fallax* recovered from fermented vegetables in combination with chitosan present a powerful inhibitory effect against three cruciferous vegetable diseases, including cabbage black spot caused by *Alternaria brassicicola*, black rot caused by *Xanthomonas campestris*, and soft rot caused by *Pectobacterium caratovorum*. The LAB/chitosan mixture is also antagonistic against *Colletotrichum higginsianum*, *Sclerotium rolfsii*, and *Fusarium oxysporum f. rapae*, suggesting a broad-spectrum activity of LAB/chitosan. Futhemore, as indicating by the experiment numerous applications are more successful than a single application. A considerable reduction in the severity of the papaya dieback disease was seen after the application of the LAB combination, *Weisella cibaria* and *Lactococcus lactis* in nurseries (Taha et al., 2019). *Lactobacillus acidophilus* which was isolated from mango (*Mangifera indica* L.) had an inhibitory action of more than 40% against post-harvest anthracnose caused by *C. gloeosporioides*. Evaluation *in vitro* demonstrated that the isolates produced antifungal chemicals as well as lytic enzymes as a mechanism of antagonism against the fungus (Fenta and Kibret, 2021).

LAB has been demonstrated in studies to have a variety of plant growth-stimulating properties to enhance the availability of nutrients to its host plants (**Table 1**), allowing them to deal with stress and inhibit plant nematodes (Amprayn et al., 2016; Ibrahim et al., 2022). According to research by Strafella et al. (2021), sixteen LAB strains evaluated in experimental settings were able to solubilize a significant amount of phosphate, and the findings corresponded to strains of *Enterococcus* sp. isolated by Mussa et al. (2018). Plant development can be promoted directly by increasing mineral and nutrient intake or indirectly by modulating plant hormones such as indole-3-acetic acid (IAA), cytokinin, and ethylene. In this case, LAB has also been demonstrated to produce indole-3-acetic acid (IAA), plant hormones that stimulate the rapid development of plants (Mohite, 2013). Three isolates from the aerial sections of the pomegranate plant that were identified as *Leuconostoc* sp. and *Lactobacillus* sp. were tested for plant growth-boosting properties by Abhyankar et al. (2022). Besides demonstrating antifungal activity against *Fusarium* sp., isolates of *Lactobacillus* sp. also exhibited 1-Aminocyclopropane 1-carboxylic acid (ACC) deaminase activity, with LAB isolate GYP3 exhibiting the highest level. This enzyme is necessary to reduce ethylene to non-toxic levels in order to protect the plants. It was also discovered that the isolate GYP3 produced indole-3-acetic acid (IAA) and Gibberellin, both of which aid in root elongation and flowering. Exopolysaccharide (EPS) was also produced by all three isolates. An oriental melon (*Cucumis melo* L.) rhizosphere LAB strain, *Enterococcus faecium* LKE12, was investigated in gibberellin (GA)-deficient rice dwarf mutant (*waito-C*) and a normal GA biosynthetic rice cultivar for its plant growth-promoting capacity (Hwayongbyeo) (Lee et al., 2015). Both regular and dwarf rice cultivars benefited greatly from *E. faecium* LKE12’s ability to secrete a wide variety of GA and IAA, which increased the shoot length and biomass of the plants, indicating a beneficial interaction between *E. faecium* LKE12 and plants. Isolates of *Lactobacillus* spp. L5, L3, and L2N found in traditional Vietnamese Nem chua exhibited Indole-acetic acid (IAA) synthesis, P-solubilization, and biofilm development (Nguyen et al., 2021). Peanut seed treatment with the same mixed bacterial cultures improved seed germination and vigor index when compared to untreated control seeds and those treated with fungicide. Those that were treated with LAB grew in both height and total fresh weight by 22.4% and 99.6%. Greenhouse and field evaluation by Shrestha et al. (2014) reported that due to its ability to secrete a significant amount of IAA, LAB strains KLF01 and KPD03 outperformed LAB strain KLC02 in terms of growth promotion, whereas KLC02 outperformed KLF01 and KPD03 in the field. Environmental conditions, root colonization, competition, and the synthesis of antagonistic metabolites are just some of the abiotic and biotic elements that could explain why greenhouse and field testing produce different results. Growth-promoting effects of several other LABs were also observed on cucumber and tomato seedlings (Lutz et al., 2012).

Effective Microorganisms (EM) consortiums are known to consist yeast, mould fungus, LAB, photosynthetic bacteria, actinomycetes, and other microorganisms. Compost incorporated with EM has been found to boost yields and nutrient uptake in a variety of crops (Javaid, 2011; Lamont et al., 2017). Fermented compost products based on lactic acid bacteria also improve soil fertility, soil structure, and aeration, neutralize alkalinity, and enhance moisture retention. Bokashi fertilizer, a traditional type of fertilizer often used in Japan, is an example of this EM-inoculated compost. Maki et al. (2021) identified 3-phenyllactic acid (PLA), a root-promoting compound from Bokashi fertilizer. PLA is a significant organic acid generated by many bacteria, particularly LAB, as a catabolic result of phenylalanine *via* phenylpyruvic acid (PPA) and has been shown to be biologically active as a plant root promoter. Recent study by Maki et al. (2022) found that PLA stimulated the auxin signalling system and influenced root development in Arabidopsis. PLA promoted lateral root density while decreasing primary root growth in Arabidopsis and elevated the expression of the auxin response marker gene *IAA19* in roots. PLA’s auxin-like activity was clearly reduced in the auxin signalling mutant, *tir1-1 afb2*, indicating that PLA regulates root development *via* the auxin signalling pathway. In a pot experiment by Javaid (2011), adding lactic acid bacteria to farmyard manure boosted rice (*Oryza sativa* L.) root and shoot growth, but not in NPK-amended soil. *Lactococcus lactis* isolated from organic soil was also found to promote plant growth in cabbage. (Somers et al., 2007). Previously, it was believed that LAB had almost minimal iron (Fe) requirement and did not produce siderophores. However, the genomes of two vegetable-isolated *Lactococcus lactis* strains isolated by Shrestha et al. (2014) revealed non-ribosomal peptide pathways, indicating the ability of LABs to produce siderophores. Further research by Jaini et al. (2022) revealed the synthesis of ammonia and siderophores, as well as the solubilization of phosphate, resulting in an increase in the dry weight of the shoot and root of papaya plants by the endophytic LAB identified in the work of Taha et al. (2019).

### 4.2 Alleviating abiotic stress in plant

Plant development can be stunted by a variety of abiotic stresses, such as flooding, dehydration, high temperatures or salt levels, the presence of toxic metals, and exposure to organic pollutants. Under abiotic stimuli, the intracellular redox balance of plants is upset which results in the production of reactive oxygen species (ROS). As a result, the plant’s enzymatic and non-enzymatic antioxidants are activated to withdraw the effects of ROS. In a condition of drought or dehydration, plant biosynthesis of nitric oxide (NO) increases in order to reduce the effects of oxidative stress. It has been observed that root treatment of wheat seedlings with *L. plantarum* 8P-A3 managed to alleviate oxidative stress during dehydration (Yarullina et al., 2014). Increases in total integral antioxidant capacity (IAC) and catalase activity indicate that NO has a role in the stress-limiting activities of lactobacilli by mitigating the deleterious effects of dehydration. Excessive salt in the soil causes ion imbalance and toxicity in plants. Plants respond to salinity stress by synthesizing polyamines and osmolytes, activating defense systems, blocking the deposition of reactive oxygen species, and controlling the transfer of ions. To counter salt-induced oxidative stress, *Swertia chirayita* inoculated with *L. plantarum* demonstrates better salinity stress tolerance by adopting more energy-efficient defensive mechanisms and efficiently partitioning carbon flow between primary and secondary metabolism (Phoboo et al., 2016). Despite the complexity of plant stress response networks still not being fully understood, LAB treatment can somehow manage to improve the stress response of plant.

## 5 Limitations, challenges, and the way forward

Similar to other types of BCAs, LAB also has its limitations and challenges when it comes to application. Currently, evidence linking LAB antagonism *in vitro* to actual pathogen control in the field is still scarce. Its basic limitation of use in agricultural applications, as with other kinds of BCA, is the capacity to survive and produce sufficient amounts of bioactive compounds in suitable circumstances. This could be overcome by selecting or designing strains through biotechnology that can flourish in the phytomicrobiome, enhacing cultures with necessary nutrients or protective carriers, and reapplying cultures to maintain a large number of viable cells. Anyhow, these methods are complicated and will take a long time. Transgenic strains with diverse modes of action can be developed using biotechnology to improve strains with desirable features such as simplicity of formulation, stability, or extraordinary suitability for plant colonization. Another alternative is to use a LAB strain more often in places that are better for its growth, like fruits, flowers, and soils with a lot of organic matter. This strategy has been effective in preventing and eradicating floral diseases that affect rosaceous tree crops (Bonaterra et al., 2014) and has promising results against postharvest infections as well (Trias et al., 2008).

The production of bioactive substances could also be accomplished through the use of LAB that has been grown in bioreactors under optimum conditions. Previous studies by Omer et al. (2009) and Limanska et al. (2015a); Limanska et al., 2015b) have shown that the metabolites produced by LABs are responsible for their activity, and the method of isolating and purifying this metabolite has been successfully applied (Maki et al., 2021). Even though LAB can endure a wide range of environmental stresses, they have specific dietary needs to thrive. Researchers have looked into how sugar beet and sweet potato processing wastes can be used to make industrial LAB media (Krzywonos and Eberhard, 2011; Hayek et al., 2013), but more consistent LAB medium is still needed to make industrial LAB culture last longer. It is also important to take precautions when planning the establishment of mixed consortia LAB with other PGPM groups to prevent incompatibilities. Nanomaterials, which have been effectively used in industries like energy, medicine, and electronics, are a newer avenue of nanotechnology being investigated and implemented in agriculture (Cruz-Luna et al., 2021). Successful applications of metal nanoparticles (M-NPs) such as silver (Ag), iron (Fe), copper (Cu), zinc (Zn), and selenium (Se) have been reported in the suppression of several phytopathogens as well as promoting plant growth in agriculture (Consolo et al., 2020; Akpinar et al., 2021). However, the chemical and physical processes utilized to create M-NPs can be both expensive and potentially hazardous to human health and the environment. As a result, ‘green’ synthesis is leading the way in this emerging discipline by exploring the viability of microorganisms and plants as nanofactories. Green synthesis of M-NPs has the benefits of being environmentally friendly, cost-effective, non-toxic, quick and reliable, stable and sustainable, with low polydispersity, scalability, and biocompatibility. Several studies have recently brought attention to the promising nanobiotechnological applications of LAB in the synthesis of intracellular and extracellular M-NPs (Alam et al., 2020; Aziz Mousavi et al., 2020; Ghosh et al., 2022). This will pave the way for further research into the role of this bacterial group in facilitating plant growth and controlling phytopathogens.

Despite its history of safe usage and “GRAS” status, the safety of the chosen LAB must be assured before industrial application to prevent having an impact on the biodiversity of the ecosystem or causing diseases in humans, animals, or plants. For example, Linares-Morales et al. (2020) reported encouraging results of *E. faecium* against post-harvest pathogens, however, further evaluation of the strains’ safety is necessary as some of the Enterococcus strains can potentially carry harmful genes (Venegas-Ortega et al., 2020). The increased efficiency of genome analysis over the past decade allows screening on the safety of LAB strains by assessing genes related to drug resistance, virulence, and pathogenicity and determining whether the related genes can be transmitted horizontally (Rodríguez-Serrano et al., 2018; Toropov et al., 2020; Zhou et al., 2021).

## 6 Conclusion

LAB strains can stimulate crop production in a number of ways, including functioning as a BCA, increasing the availability of nutrients, mitigating the effects of biotic and abiotic stressors, and stimulating plant growth directly. Its GRAS status and extensive history in food research make them ideal for use in crop protection. Although LABs are ubiquitous in the phytomicrobiome, their potential roles as BCAs and promoters of plant development have been generally disregarded. Evidence from the past and present points to the fact that LAB has the ability to serve as renewable and safe agricultural inputs that can aid in the control of plant diseases and the promotion of plant growth. However, more LAB studies are needed and should focus on its biocontrol efficiency under field conditions as well as LAB bioproduction and formulations. The integration of LAB as biocontrol agents that could be used with other biocontrol techniques in an integrated control program would be a viable way to increase efficacy against phytopathogens and help solve the challenges to achieving sustainable food security.

## Author contributions

Conceptualization, NJ and KC. Methodology, NJ and KC. Validation, RJ and KP. formal analysis, NJ. Investigation, NJ. Resources, KC. Writing—original draft NJ. Writing—review and editing, RJ and KC. Visualization, NJ & KC. Supervision, RJ & KC. All authors contributed to the article and approved the submitted version.
